# Middle East Respiratory Syndrome in 3 Persons, South Korea, 2015

**DOI:** 10.3201/eid2111.151016

**Published:** 2015-11

**Authors:** Jeong-Sun Yang, SungHan Park, You-Jin Kim, Hae Ji Kang, Hak Kim, Young Woo Han, Han Saem Lee, Dae-Won Kim, A-Reum Kim, Deok Rim Heo, Joo Ae Kim, Su Jin Kim, Jeong-Gu Nam, Hee-Dong Jung, Hyang-Min Cheong, Kisoon Kim, Joo-Shil Lee, Sung Soon Kim

**Affiliations:** Korea Centers for Disease Control and Prevention, Cheongju, South Korea

**Keywords:** Middle East respiratory syndrome, MERS, MERS-CoV, South Korea, viruses, respiratory infections

## Abstract

In May 2015, Middle East respiratory syndrome coronavirus infection was laboratory confirmed in South Korea. Patients were a man who had visited the Middle East, his wife, and a man who shared a hospital room with the index patient. Rapid laboratory confirmation will facilitate subsequent prevention and control for imported cases.

Middle East respiratory syndrome (MERS) is characterized by mild-to-severe respiratory distress and is caused by a novel coronavirus (MERS-CoV) (*1*). Since its first identification in 2012, MERS-CoV infection has been reported for 1,413 persons from 26 countries; case-fatality rate is 40.92% (*2*). We describe an outbreak comprising 3 laboratory-confirmed cases of MERS-CoV infection in South Korea (*3*–*5*).

## The Study

**Figure 1:**
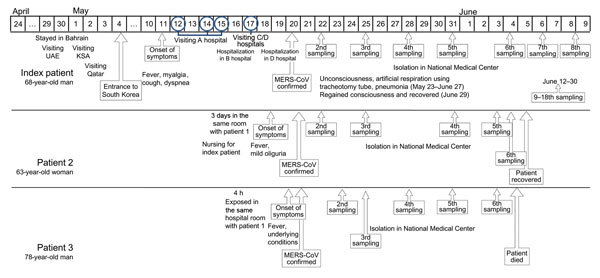
Timeline of events for patients infected with Middle East respiratory syndrome coronavirus (MERS-CoV). The laboratory diagnostic methods used for molecular detection of MERS-CoV RNA were multiplex MERS-CoV real-time reverse transcription PCRs targeting an upstream MERS-CoV envelope protein gene and an open reading frame 1a gene (*8*,*9*). KSA, Kingdom of Saudi Arabia; UAE, United Arab Emirates.

In accordance with national MERS control guidelines in South Korea (*6*), specimens are collected from hospitalized patients suspected of having MERS on the basis of epidemiologic history linked to the Middle East; these specimens are then transferred to the Korea National Institute of Health for examination. The index patient, a 68-year-old man engaged in farming-related business, reported that he had traveled to Bahrain on April 24, 2015, the United Arab Emirates on April 29–30, Saudi Arabia on May 1–2, and Qatar on May 2–3 before returning to South Korea on May 4 (Figure 1). While in these countries, he was not exposed to any patients, health care facilities, or animals (including camels and bats) or their excreta. On May 11, the patient experienced chills and a fever (>37°C), and an allopathic medicine was prescribed when he first visited a local clinic. However, his symptoms worsened (temperature >38°C, myalgia, cough, and dyspnea), and after he had visited 4 hospitals (hospitals A–D, in or around Seoul), he was admitted to a general hospital (hospital D, Seoul, South Korea) on May 18. A nasopharyngeal aspiration specimen was collected for MERS-CoV laboratory testing on May 19**.**

Sputum samples from 2 persons who had been in contact with the index patient were also tested for MERS-CoV. Patient 2 was the 63-year-old wife of the index patient; she had had physical contact with him while caring for him during the 3 days of hospitalization. Fever (38°C) and slight oliguria developed in patient 2 on May 19. The other contact, patient 3, was a 78-year-old man who had chronic obstructive pulmonary disease, asthma, and cholangiocarcinoma and who had shared a hospital room with the index patient and had been within 2 meters from him for 4 hours on May 16. Fever (37.8°C) and respiratory symptoms developed in patient 3 on May 20. In the hospital room, the index patient did not undergo any aerosol-generating procedures, but a severe cough developed. The same health care workers cared for the index patient and patient 3.

Laboratory diagnostic methods were performed according to World Health Organization guidelines for molecular detection of MERS-CoV (*7*–*9*). To check for contamination derived from the positive control, we designed and synthesized the MERS-CoV real-time reverse transcription PCR (rRT-PCR)–positive transcripts for an upstream MERS-CoV envelope protein gene (upE) and the open reading frame 1a (ORF1a) gene containing 50 bp of a foreign gene (centipede). 

Initially, nasopharyngeal samples from the index patient were positive for MERS-CoV by multiplex rRT-PCR. Sputum samples from patients 2 and 3 were also positive, supporting a diagnosis of MERS-CoV infection. Multiplex rRT-PCR results for upE and ORF1a were positive (Table 1). According to rRT-PCR, the respiratory samples from the 3 patients were negative for 5 other human coronaviruses (SARS-CoV and human CoV-229E, -OC43, -NL63, and -HKU1) and 7 viruses that cause acute respiratory infection (influenza virus A and B; human adenovirus; bocavirus; human parainfluenza virus types 1, 2, and 3; respiratory syncytial virus A and B; human rhinovirus; human metapneumovirus).

**Table 1:** Real-time reverse transcription PCR results for 2 patients infected with Middle East respiratory syndrome coronavirus, South Korea, 2015*

Sample	Index patient (day 9 after illness onset)			Wife of index patient (day 2 after illness onset)	
	upE, C_t_	ORF1a, C_t_		upE, C_t_	ORF1a, C_t_
Sputum	18.61	19.32		26.23	26.63
Nasal swab	29.34	29.58		ND	36.64
Nasopharyngeal aspirate	29.35	31.45		NT	NT
Serum	34.81	35.50		ND	ND
Urine	ND	ND		34.34	ND

*C_t_, cycle threshold; ND, not detected; NT, not tested; ORF1a, open reading frame 1a gene; upE, upstream envelope protein gene.

For the index patient, MERS-CoV RNA was detectable in sputum, throat swab, and serum samples but not in a urine sample collected 9 days after symptom onset (Table 1). The viral load, indicated by cycle threshold values, was high in the lower respiratory tract sample but almost undetectable in the throat swab and serum samples. After sequential sampling repeated every 2–5 days, MERS-CoV RNA was detected in sputum until 44 days after symptom onset, although viral RNA was inconsistently detected and patterns of viral load fluctuated (Table 2). Other than initial fever (>37°C), clinical features differed for all 3 patients. The index patient had respiratory symptoms with cough, dyspnea, and myalgia. Patient 2 did not have a relevant medical history and showed mild symptoms. Patient 3 had underlying concurrent conditions and died 16 days after confirmation of MERS-CoV infection (Figure 1).

**Table 2:** Real-time reverse transcription PCR results for sputum samples serially collected from 3 patients infected with Middle East respiratory syndrome coronavirus, South Korea, 2015*

Date	Patient 1 (index patient)				Patient 2†				Patient 3‡		
	Days after illness onset	upE, C_t_	ORF1a, C_t_		Days after illness onset	upE, C_t_	ORF1a, C_t_		Days after illness onset	upE, C_t_	ORF1a, C_t_
May 20	9	18.61	19.32		2	26.23	26.63		1	28.10	28.65
May 22	11	25.24	25.67		5	35.53	35.10		3	24.67	25.04
May 25	14	26.48	27.99		11	25.20	26.10		5	26.37	25.53
May 28	17	31.23	32.05		14	32.58	34.80		8	ND	35.94
May 31	20	36.94	ND		17	ND	ND		11	26.30	28.01
Jun 4	24	ND	36.27		18	ND	ND		14	ND	ND
Jun 6	26	33.92	36.70		19	Discharged			15	Died	
Jun 8	29	36.50	ND								
Jun 9	30	36.90	37.46								
Jun 12	33	ND	ND								
Jun15	36	ND	ND								
Jun 16	37	35.46	ND								
Jun 17	38	ND	ND								
Jun 22	43	ND	ND								
Jun 23	44	32.97	36.31								
Jun 26	47	ND	ND								
Jun 29	50	ND	ND								
Jun 30	51	ND	ND								

*C_t_, cycle threshold; ND, not detected; ORF1a, open reading frame 1a gene; upE, upstream envelope protein gene. Blank cells indicate not applicable.  †Wife of index patient. ‡Shared hospital room with index patient.

**Figure 2:**
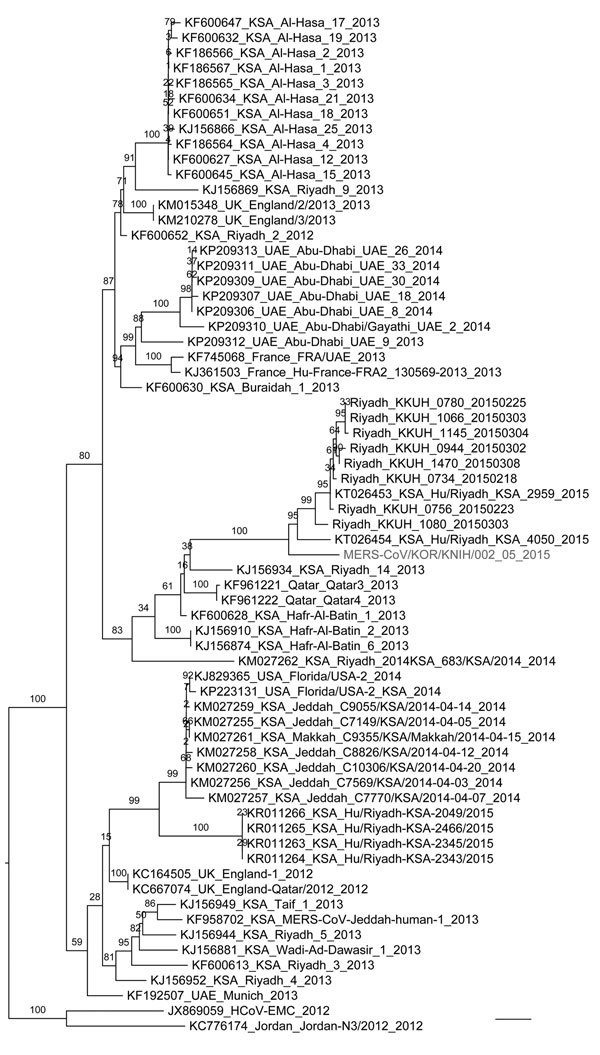
Phylogenetic tree comparing complete genome nucleotide sequences of Middle East respiratory syndrome coronavirus (MERS-CoV) isolate from South Korea (KOR/KNIH/002_05_2015) with those of 67 reference MERS-CoVs (GenBank database). The tree was constructed by using the general time reversible plus gamma model of RAxML version 8.8.0 software (*10*) and visualized by using FigTree version 1.4.2 (http://tree.bio.ed.ac.uk/software/figtree). RAxML bootstrap values (1,000 replicates) are shown above the branches. Bootstrap values >75 are shown on the branches. The MERS-CoV strain from South Korea is shown in gray. National Center for Biotechnology Information accession numbers are shown before each taxon name. The unit of branch length is the number of substitutions per site. Scale bar indicates 4 × 10^–4^ nucleotide substitutions per site.

Virus isolation on Vero cells was attempted for each respiratory specimen from the 3 patients. The culture supernatant after inoculation was serially assessed for virus growth by using rRT-PCR and was used for blind passages every 3–7 days after inoculation. After 3 blind passages, cytopathic effect was observed, and we isolated the MERS-CoV strain from South Korea (KOR/KNIH/002_05_2015) from the Vero cells after inoculation by using the sputum from patient 2. We constructed a phylogenetic tree by using the general time reversible plus gamma model of the RAxML version 8.8.0 software (*10*) and FigTree version 1.4.2 (http://tree.bio.ed.ac.uk/software/figtree) and by using complete genomes of the MERS-CoV isolate from South Korea (GenBank accession no. KT029139) and 67 reference MERS-CoVs (Figure 2).

## Conclusions

Because, to our knowledge, cases of MERS-CoV infection in South Korea have not been reported, we had to establish laboratory testing protocols to overcome vulnerabilities in the absence of appropriate epidemiologic support (i.e., generate positive controls to check for contamination and repeat testing). Positive controls containing foreign genes have been generated to check for laboratory contamination. For patients with an unclear exposure history (such as the index patient) and for patients with short exposure durations and unusual clinical symptoms (such as patients 2 and 3), it would be useful if the positive results of rRT-PCR could be confirmed through agarose gel electrophoresis to exclude contamination from the positive control (*3*,*4*).

The index patient had no history of potential exposure to camels, bats, or their excreta; to symptomatic persons; or to health care workers during his trip to the Middle East, including Saudi Arabia. Although the source of infection for the index patient is unclear, phylogenetic analysis of the whole viral genome showed that the isolate from South Korea was closely related to the MERS-CoV strains isolated in Saudi Arabia in 2015.

Because the index patient initially concealed his travel history to Saudi Arabia, United Arab Emirates and Qatar, MERS-CoV infection was not considered and the patient was not isolated until MERS-CoV infection was suspected 7 days after symptom onset. Meanwhile, other patients and health care workers had multiple opportunities for exposure to the index patient (*3*–*5*). The 2 contacts reported here had each been exposed to the index patient. Patient 3 was probably infected via droplet transmission in the hospital room. The hospital room, originally built for 6 persons, had been divided into 2 rooms and lacked ventilation. Furthermore, an air conditioning unit cycled the air in the room with the door and window closed. Thus, poor ventilation might have played a major role in droplet transmission. Detection of MERS-CoV RNA in the respiratory tract varies up to day 33 (*11*–*13*). In this study, virus was detected in the respiratory tract, inconsistently, for up to 44 days. 

Development of effective preventive measures for the MERS-CoV prevention will require systemic and prospective studies associated with viral shedding and use of specimens in addition to those obtained from the respiratory tract to define the kinetics of MERS-CoV. Rapid detection of MERS-CoV, using multiplex rRT-PCR to detect upE and ORF1a genes, would be helpful for countries outside the Arabian Peninsula.
